# Ferroptosis in kidney disease: a bibliometric analysis from 2012 to 2024

**DOI:** 10.3389/fphar.2024.1507574

**Published:** 2025-01-13

**Authors:** Yuxin Hu, Jingyi Tang, Hanzhang Hong, Yexin Chen, Beibei Ye, Ziheng Gao, Gegongming Zhu, Lin Wang, Weijing Liu, Yaoxian Wang

**Affiliations:** ^1^ Dongzhimen Hospital, Beijing University of Chinese Medicine, Beijing, China; ^2^ Beijing University of Chinese Medicine, Beijing, China; ^3^ Renal Research Institution of Beijing University of Chinese Medicine, Dongzhimen Hospital Affiliated to Beijing University of Chinese Medicine, Beijing, China; ^4^ Key Laboratory of Chinese Internal Medicine of Ministry of Education and Beijing, Dongzhimen Hospital Affiliated to Beijing University of Chinese Medicine, Beijing, China; ^5^ Henan University of Chinese Medicine, Zhengzhou, Henan, China

**Keywords:** ferroptosis, kidney disease, kidney, bibliometric analysis, hotspots, trends

## Abstract

**Background and aims:**

Ferroptosis, a novel concept of programmed cell death proposed in 2012, in kidney disease, has garnered significant attention based on evidence of abnormal iron deposition and lipid peroxidation damage in the kidney. Our study aim to examine the trends and future research directions in the field of ferroptosis in kidney disease, so as to further explore the target or treatment strategy for clinical treatment of kidney disease.

**Material and Methods:**

A thorough survey using the Web of Science Core Collection, focusing on literature published between 2012 and 2024 examining the interaction between kidney disease and ferroptosis was conducted. VOSviewer, CiteSpace, and Biblioshiny were used for in-depth scientometric and visualized analyses.

**Results:**

From 2012 to 2024, a total of 2,244 articles met the inclusion criteria for final analysis. The number of annual publications in this area of study showed a steady pattern at the beginning of the decade. The top 3 journals with the highest publication output were *Renal Failure*, *Oxidative Medicine And Cellular Longevity*, and *Biomedicine & Pharmacotherapy*. China and the United States had the highest number of publications. Central South University and Guangzhou Medical University as the most active and influential institutions. Documents and citation analysis suggested that Andreas Linkermann, Jolanta Malyszko, and Alberto Ortiz are active researchers, and the research by Scott J. Dixon and Jose Pedro Friedmann Angeli, as the most cited article, are more important drivers in the development of the field. Keywords associated with glutathione, lipid peroxidation, and nitric oxide had high frequency in the early studies. In recent years, however, there has been a shift towards biomarkers, inflammation and necrosis, which indicate current and future research directions in this area.

**Conclusion:**

The global landscape of the ferroptosis research in kidney disease from 2012 to 2024 was presented. Basic research and mechanism exploration for renal fibrosis and chronic kidney disease may be a hot spot in the future.

## 1 Introduction

Chronic kidney disease (CKD) is a common chronic progressive disease characterized by abnormal renal structure and dysfunction caused by various potential diseases. The global prevalence of CKD is rapidly increasing, leading to an increased risk of cardiovascular events, renal failure, and death (Gansevoort et al., 2013). Kidney is an important organ involved in iron filtration and reabsorption, which plays an important role in regulating iron metabolism. The kidney is rich in mitochondria and has a high oxygen uptake, which is susceptible to the imbalance of oxidation and antioxidant systems (Ho and Shirakawa, 2022). In addition, CKD is associated with lipid metabolism disorders and lipid accumulation. The accumulation of lipids can activate the innate immune system, promote inflammatory fibrosis, cause mitochondrial damage, and drive CKD progression (Mitrofanova et al., 2023). Therefore, the key role of ferroptosis in the occurrence and development of kidney disease has been paid more and more attention, which may become an emerging target for clinical treatment and related drug development.

Ferroptosis, a programmed cell death, is driven by iron-dependent lipid peroxidation, which was discovered and proposed in 2012 and plays an important role in a wide range of biological backgrounds from development to aging, immunity, cancer and so on (Stockwell, 2022). Different from apoptosis and other forms of cell death, ferroptosis is characterized by the accumulation of intracellular iron ions, which destroys the balance of intracellular lipid peroxidation system, leading to lipid peroxidation and ultimately cell death (Stockwell et al., 2020). The key points of ferroptosis regulation are cystine transport, fatty acid synthesis and iron transport. Cystine is transported into cells through the Xc-system and converted into cysteine, which forms reduced glutathione (GSH) together with glutamic acid and glycine. GSH depletion, and decreased cellular antioxidant capacity lead to lipid peroxidation and reactive oxygen (ROS) accumulation, eventually resulting in ferroptosis (Mao et al., 2024). According to the recommendations of the Nomenclature Committee on Cell Death in 2018, ferroptosis is defined as a regulatory cell death caused mainly by glutathione peroxidase 4 (Gpx4) disorders regulating intracellular oxidative imbalance (Galluzzi et al., 2018). As a key enzyme in the development of ferroptosis, Gpx4 promotes the conversion of phospholipid hydroperoxides into less harmful lipid alcohols, thereby protecting cells from damage caused by lipid peroxidation. Inhibition or reduction of Gpx4 activity will increase the susceptibility of cells to ferroptosis (Xie et al., 2023). In recent years, studies have found that oxidative stress, drugs and other factors can induce ferroptosis in a variety of diseases, including neurological diseases, cancer, cardiovascular diseases and kidney diseases (Lei et al., 2020; Fang et al., 2023; Guo et al., 2023). An increasing number of reports have also linked ferroptosis to kidney disease. Some changes related to ferroptosis have been found in the kidneys of patients with kidney disease, such as oxidative stress, excessive iron accumulation and accumulation of lipid peroxidation products, and iron accumulation has been found to drive the development of fibrosis (Maus et al., 2023). Therefore, a comprehensive understanding of the pathogenesis and regulatory pathways of ferroptosis is crucial for the development of more effective treatment strategies. The regulation of ferroptosis as a means to influence the onset and progression of related diseases has emerged as a prominent research focus. However, the functional alterations and precise molecular mechanisms underlying ferroptosis require further investigation.

In recent years, there has been a significant increase in research examining the role of ferroptosis in kidney disease, with recent reviews addressing various dimensions of this concept (Feng et al., 2022; Wang et al., 2023). However, a comprehensive analysis of the present situation and future trajectory of the subject, as well as an objective delineation of the research focus in this field, is currently lacking. Bibliometric analysis represents an effective method for evaluating the overall trend of a given field of study. The tool assists researchers and clinicians understand the core countries, institutions and authors, as well as the most influential node publications within a specific research domain. Additionally, it enables the identification of thematic shifts, emerging research trends and research gaps in the field (Ninkov et al., 2022). This method has been applied to various disciplines including medical research (Oh and Flaherty, 2020; Ahmad and Slots, 2021). As far as we know, the relationship between ferroptosis and neurodegenerative diseases, cardiovascular diseases and cancer has been studied by bibliometrics. However, it has yet to be applied to the study of ferroptosis and kidney diseases (Lu et al., 2023; Miao et al., 2023, p. 202; Jiang et al., 2024). Hence, we aim to reveal the current research status and development trend of ferroptosis in kidney disease through bibliometric analysis, and provide reference for future researches.

## 2 Methods

### 2.1 Data source and search strategy

Web of Science is the most comprehensive and authoritative data platform in the world, gathering high-quality research. Therefore, Web of Science Core Collection (WoSCC) was used to research related publications. In this study, we comprehensively collected all relevant publications published from 1 January2012 to 2 July2024, and constructed the search formula as follows:

#1 TS = (“ferroptosis” OR (“GSH” AND “GPX4”) OR “lipid peroxidation” OR “iron homeostasis” OR “iron metabolism”)
#2 TS = (“renal disease” OR “kidney disease” OR “kidney trouble” OR “nephropathy” OR “nephrosis” OR “nephropathic”)
#3 TS = (“nephritis” OR “renal fibrosis” OR “renal interstitial fibrosis” OR “renal damage” OR “renal failure” OR “renal insufficiency”)
#4 TS = (“chronic kidney disease” OR “acute kidney injury” OR “diabetic nephropathy” OR “iga nephropathy” OR “kidney cancer”)
#5 TS = (“renal tubular disease” OR “glomerulonephritis” OR “glomerular disease” OR “renal capsule disease” OR “glomerular capsule”)
#1 AND (#2 OR #3 OR #4 OR #5)

**FIGURE 1:**
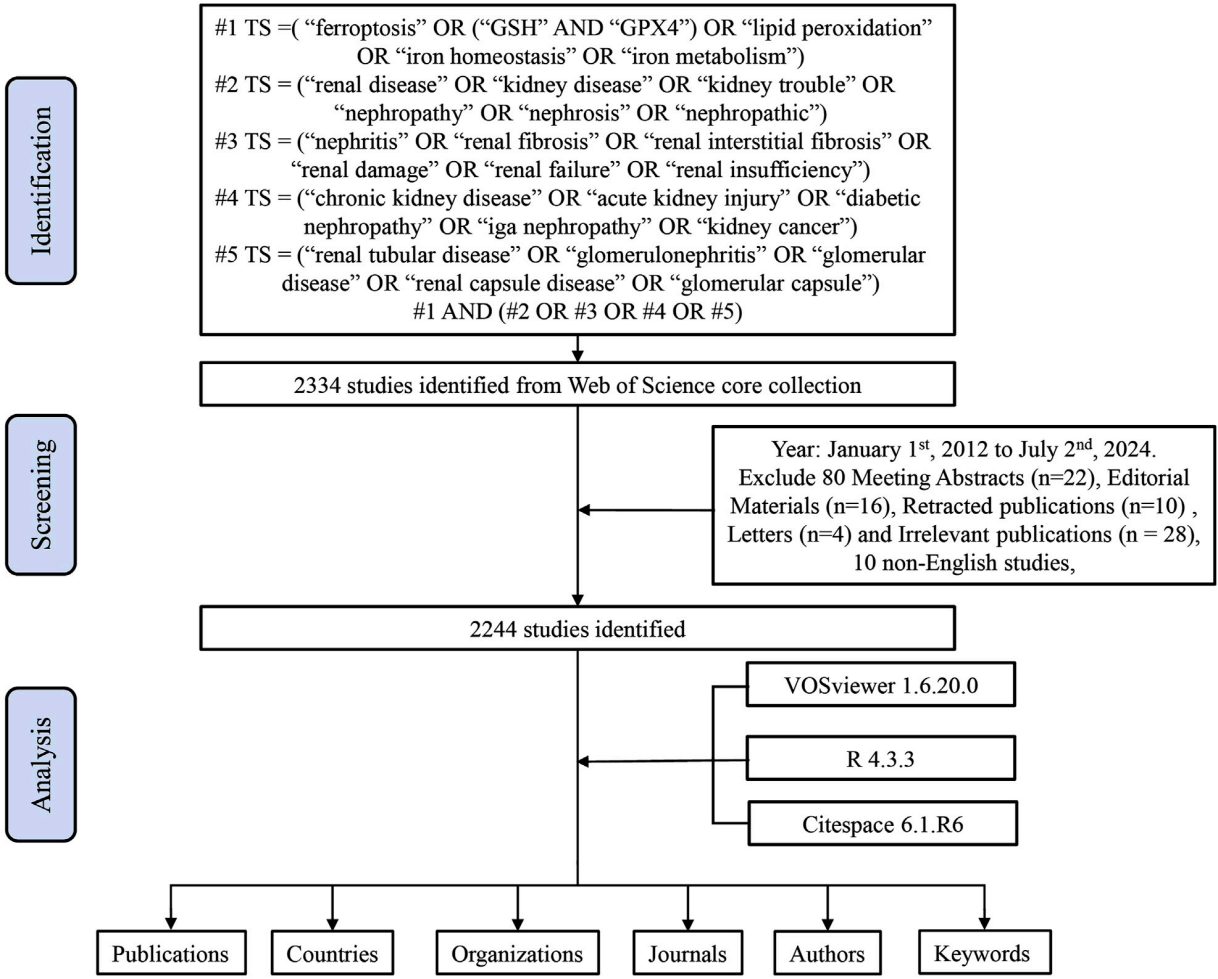
Study research process and search strategy. Only studies published between 1 January 2012 and 2 July 2024 were eligible for inclusion. This approach initially led to the identification of 2,334 studies, 10 of which were not published in English and were excluded. Subsequently, 2,244 of the remaining studies were deemed eligible for inclusion, while 80 failed to meet the designated inclusion criteria. TS, topic search.

We retrieved a total of 2,334 studies, exported complete records and cited references in plain text files, and removed 10 non-English articles and 80 Meeting Abstracts, Articles, Proceedings Papers, Editorial Materials, Corrections, Book Chapters, Early Accesses. After Letters and Retracted publications, a total of 2,244 articles were retained (Figure 1). Ferroptosis has quickly become a research hotspot since it was proposed in 2012. We have collected all the studies since the concept of ferroptosis was proposed. It is believed that the analysis of this time segment can reflect the research trend in this field, aiming to show the role of ferroptosis in the kidney from a more scientific and comprehensive perspective for other scholars.

### 2.2 Data collection and quality assessment

H index and Impact Factor (IF) were used to explain our analysis results. The H index, which is used to estimate the importance and broad impact of scientists’ cumulative research contributions, has become a well-known indicator for assessing the research influence of academic scholars (Hirsch, 2005). The H index was invented by Jorge Eduardo Hirsch, an Argentine-American professor of physics at the University of California, San Diego, in 2005. If the author’s published papers (denoted by number of papers, Np) have H references at least H times, and each of the other (Np-H) papers is cited ≤ H times, the scientist’s index is H (Hirsch, 2005). IF is often used as a measure of journal quality, and research published in journals with high IF index often represents higher academic value (Severin et al., 2023).

### 2.3 Bibliometric analysis and visualization

In this study, VOSviewer (1.6.20.0), Citespace (6.1. R6), Biblioshiny (4.1.4) package in R were used for bibliometric analysis. In addition to the VOSviewer and Citespace, ggplot2 (3.4.2) package in R language and SCImago Graphica Beta (1.0.39) were used for visualization. VOSviewer was used to analyze the distribution of publishers and authors and the frequency of cooperation, keywords, and co-citation of journals. At the same time, the data exported by VOSviewer was used for geographic visualization analysis in SCImago to analyze the cooperation between countries. Citespace was used for keyword clustering analysis, keyword timeline analysis and keyword burst analysis. In addition to the maps generated by VOSviewer and Citespace, specific data such as the number of publications, the number of citations, and the keyword burst time are visualized using ggplot2.

VOSviewer is a Java-based free software developed in 2009 by Van Eck and Waltman of the Science and Technology Research Center at Leiden University in the Netherlands. It has strong graphics capabilities, is suitable for processing large-scale data, and focuses on the visualization of bibliometric networks (van Eck and Waltman, 2010). In addition, VOSviewer supports overlay visualization maps, where the color and distance of nodes represent the distribution of nodes in two-dimensional space, i.e., time and association (van Eck et al., 2013). SCImago Graphica software does not have the functions of network node analysis, path measurement and clustering, but by combining with the visualization software of scientific text mining, it can carry out cooperative network analysis (countries, institutions, authors), keyword co-occurrence analysis and geographic visualization analysis of literature collection.

## 3 Results

### 3.1 An overview of publications

**FIGURE 2:**
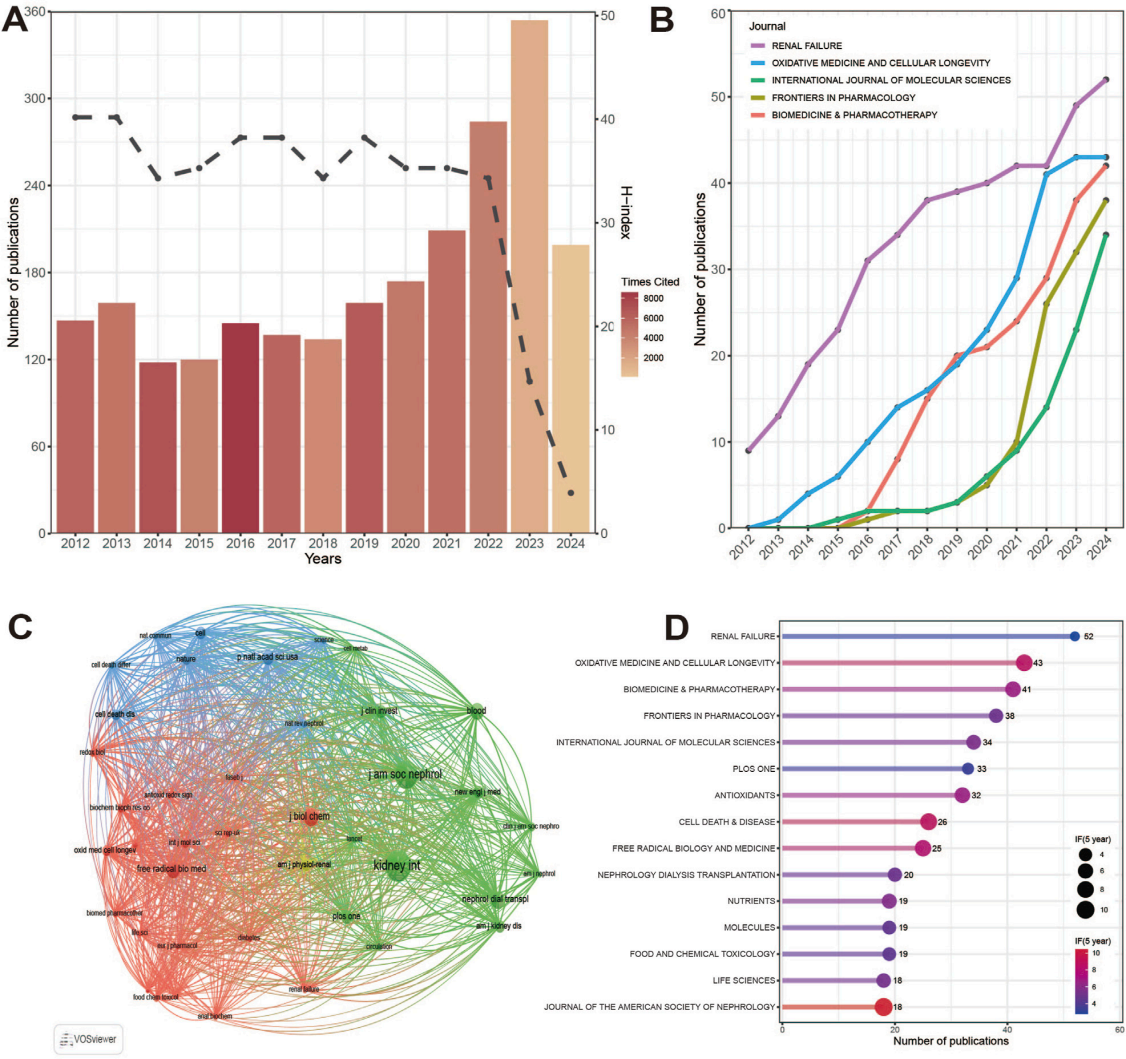
Temporal distribution map of the literature. **(A)** Global trends in annual publications, citations, and H-indexes related to ferroptosis in kidney disease 2012–2024. **(B)** The publication trend of the top five journals. **(C)** Co-citation analysis of journals of iron death publications in kidney diseases. **(D)** Top 15 journals and their IF index of publications in 2024. H-index: The Hirsch index, also known as the H-index, is used to estimate the importance and broad impact of scientists ' cumulative research contributions. IF: impact factors.

The number of publications over a period of time can reflect the speed and trend of research in this field (Qin et al., 2020). From 1 January 2012 to 2 July 2024, a total of 2,244 publications related to ferroptosis in kidney disease were identified. Figure 2A shows the number of annual publications, H index and citations in this field. Figure 2B shows the trend of the top 5 journals in 2024 publishing articles from 2012 to 2024. Figure 2C shows the results of co-citation analysis of journals. Figure 2D shows the top 15 journals published in 2024 and the corresponding Journal Impact Factor.

In general, the number of publications in the early stage (2012–2018) often fluctuates around 100–150, and has shown a relatively stable growth trend since 2019. The H index has been fluctuating between 240 and 300 and will decline in 2023. The number of citations has always remained at a high level. The above three indicators reflect the theme of ferroptosis in kidney disease. Since the concept of ferroptosis was put forward, it has quickly become a hot research direction, aroused strong research interest, and the research heat continues. In terms of the number of publications, the largest number of publications published in 2024 was *Renal Failure* (52), followed by *Oxidative Medicine and Cellular Longevity* (43) and *Biomedicine* & *Pharmacotherapy* (42). The number of publications in *Renal Failure* was always higher than that in other journals from 2012 to 2024.

### 3.2 National contributions and international collaboration

**FIGURE 3:**
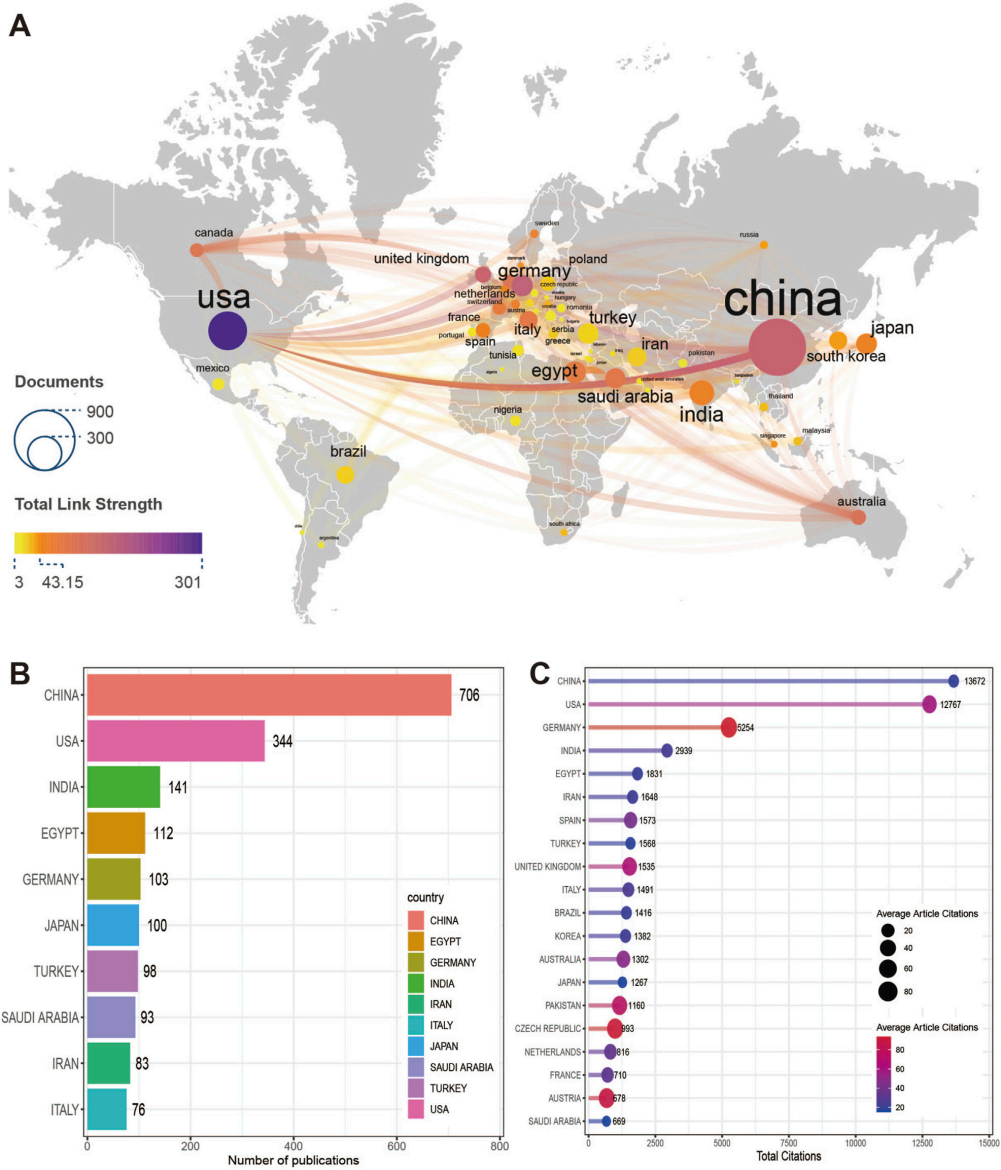
National contributions and international collaboration. **(A)** World country map. Each node represents a country. The size of the node is positively correlated with the number of published articles. The color of the node is positively correlated with the intensity of cooperation. The intensity of cooperation is quantified by the Total link strength calculated by VOSviewer. **(B)** The top 20 countries ranked by total citations. The size of the node is positively correlated with the average article citations. **(C)** The top 10 most productive countries.

A total of 89 countries have published articles and contributed to the study of ferroptosis in kidney disease. Figure 3A shows that many countries have established cooperative relations. Among them, the United States is the country with the most extensive cooperation, and has established cooperative relations with 55 countries, followed by Australia (49 countries) and the United Kingdom (46 countries). Figure 3B shows the top 20 countries cited. China also ranks first with 13,672 times, but the highest average article citation is Germany (93.80), followed by Czech Republic (90.30) and Austria (84.80). Figure 3C shows the top 10 countries with the highest number of publications, of which China is the most productive country, with 706 articles published in the past 13 years, contributing nearly a third of the articles, followed by the United States (344) and India (141).

### 3.3 Institutional research output

**FIGURE 4:**
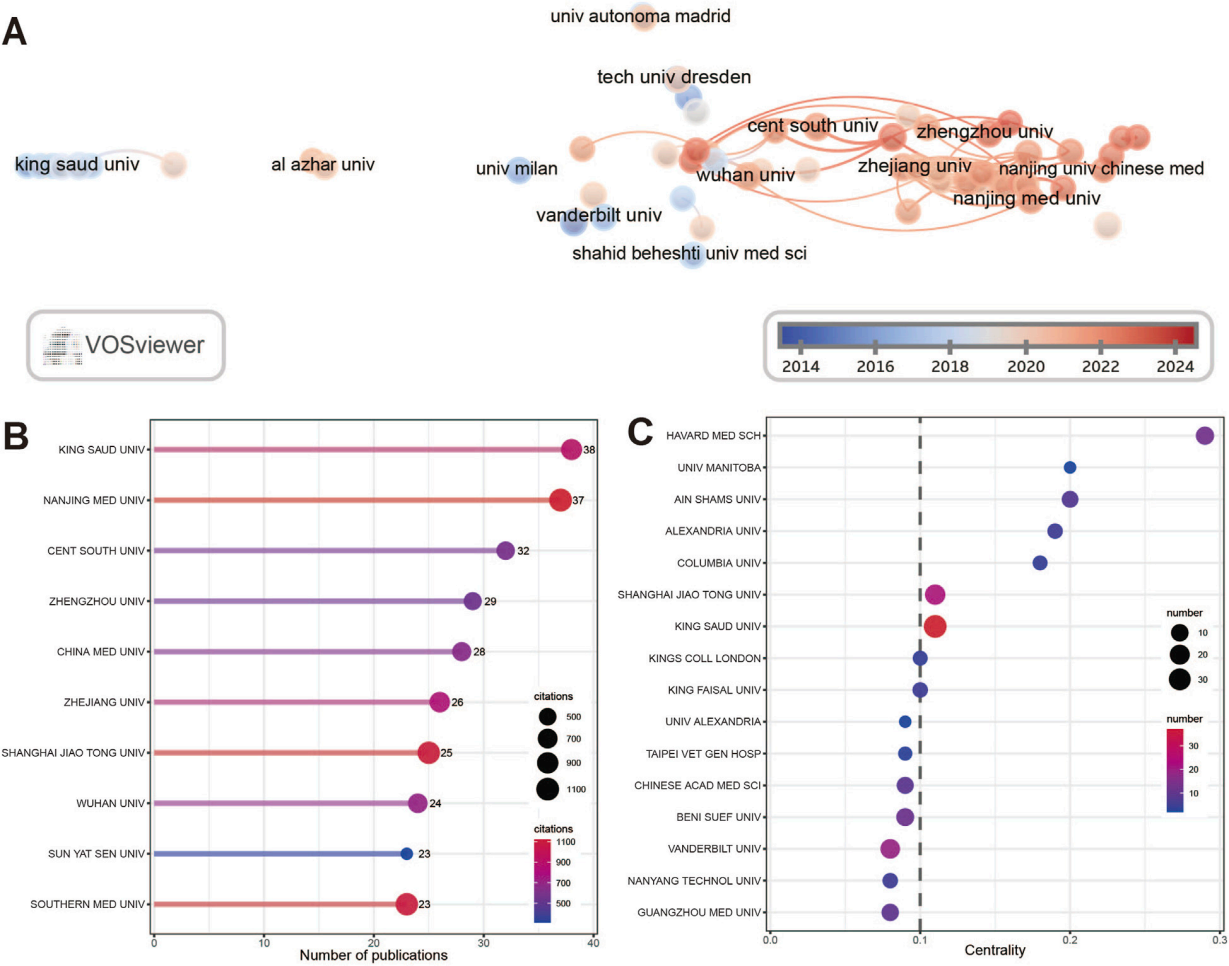
Visualization of organizations cooperation. **(A)** Visualization analysis of inter-agency cooperation. Each node represents an organization. The node size is positively correlated with the number of published articles, the node color is related to time, and the thickness of the lines between nodes is positively correlated with the frequency of cooperation. **(B)** The top 10 institutions ranked by the number of publications. The size and color of nodes are related to the number of citations of articles published by institutions. **(C)** Top 15 institutions ranked by centrality. The node size and color are positively correlated with the number of articles published by the organization.

A total of 2,795 institutions from 89 countries have published research, and the cooperation network diagram is shown in Figure 4A. The current cooperation of institutions is still limited to domestic cooperation, and the cooperation between countries is still insufficient. Encourage institutions to seek cooperation more actively to promote development in this field. In terms of the number of publications (Figure 4B), King's University of Saudi Arabia is the highest-yield institution, with 38 articles published, and the remaining 9 high-yield institutions are from China, once again indicating that China has invested a lot of energy in the study of ferroptosis in kidney diseases and is in a leading position in this field. In Figure 4C, institutions with centrality greater than or equal to 0.1 can be considered to play a key role in this field (Zhong et al., 2021). Harvard Medical School from the United States has the highest centrality (0.29), followed by Ainshams University from Egypt (0.2) and University of Manitoba from Canada (0.2).

### 3.4 Research productivity by individual authors

**FIGURE 5:**
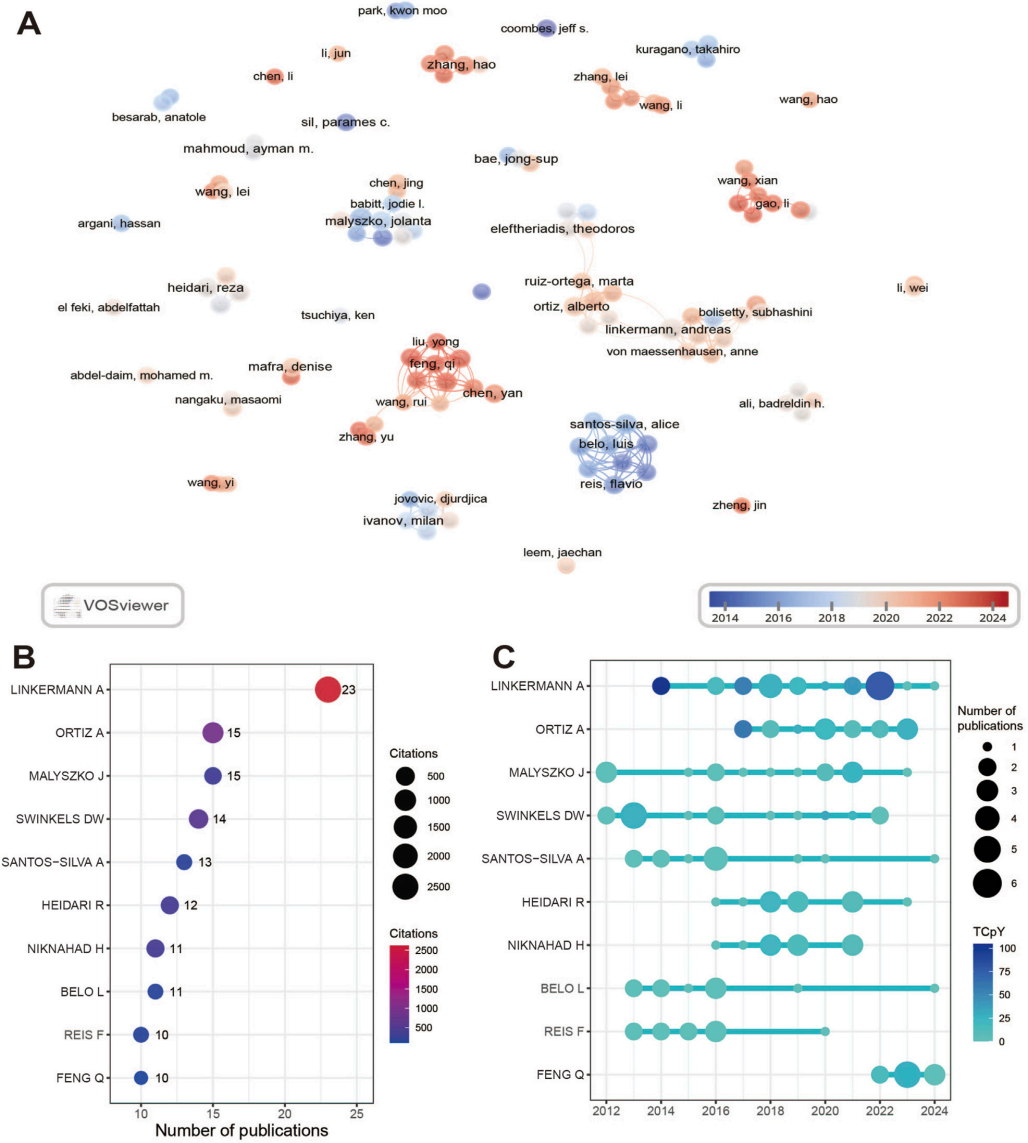
Author analysis. **(A)** Visual analysis of cooperation between authors. Each node represents an author. The size of the node is positively correlated with the number of published articles. The color of the node is related to the time. The thickness of the lines between the nodes is positively correlated with the frequency of cooperation. **(B)** The top 10 authors in the number of publications. The node size is positively correlated with the number of articles published by the author, and the node color is related to the number of citations published by the author. **(C)** The number of publications in the top 10 authors published articles in 2012–2024. Ranked from top to bottom according to the first letter of the author‘s surname, the size of the node is positively correlated with the number of publications published by the author, and the color of the node is related to the average number of citations per year. TCpY: Total citations per year.

The analysis of the authors is helpful to understand the representative researchers and core research trends in a field. The H index can explain the importance, significance and broad impact of scientists’ cumulative research contributions (Hirsch, 2005). A total of 12,551 authors have contributed to the development of this field. Figure 5A shows the frequency of cooperation between authors. It can be seen that the scope of cooperation between authors is small and the frequency is low. Authors should be encouraged to develop a broader cooperative relationship. As shown in Figure 5B, the most prolific author was Andreas Linkermann from Germany, who published 23 articles with H index as high as 60, followed by Jolanta Malyszko from Poland (15 articles with H index of 43) and Alberto Ortiz from Spain (15 articles with H index of 100). Figure 5C shows that most of the high-yield authors have published intensive articles after 2020, indicating that ferroptosis in kidney disease has attracted extensive research interest.

### 3.5 Co-cited documents and journals

**FIGURE 6:**
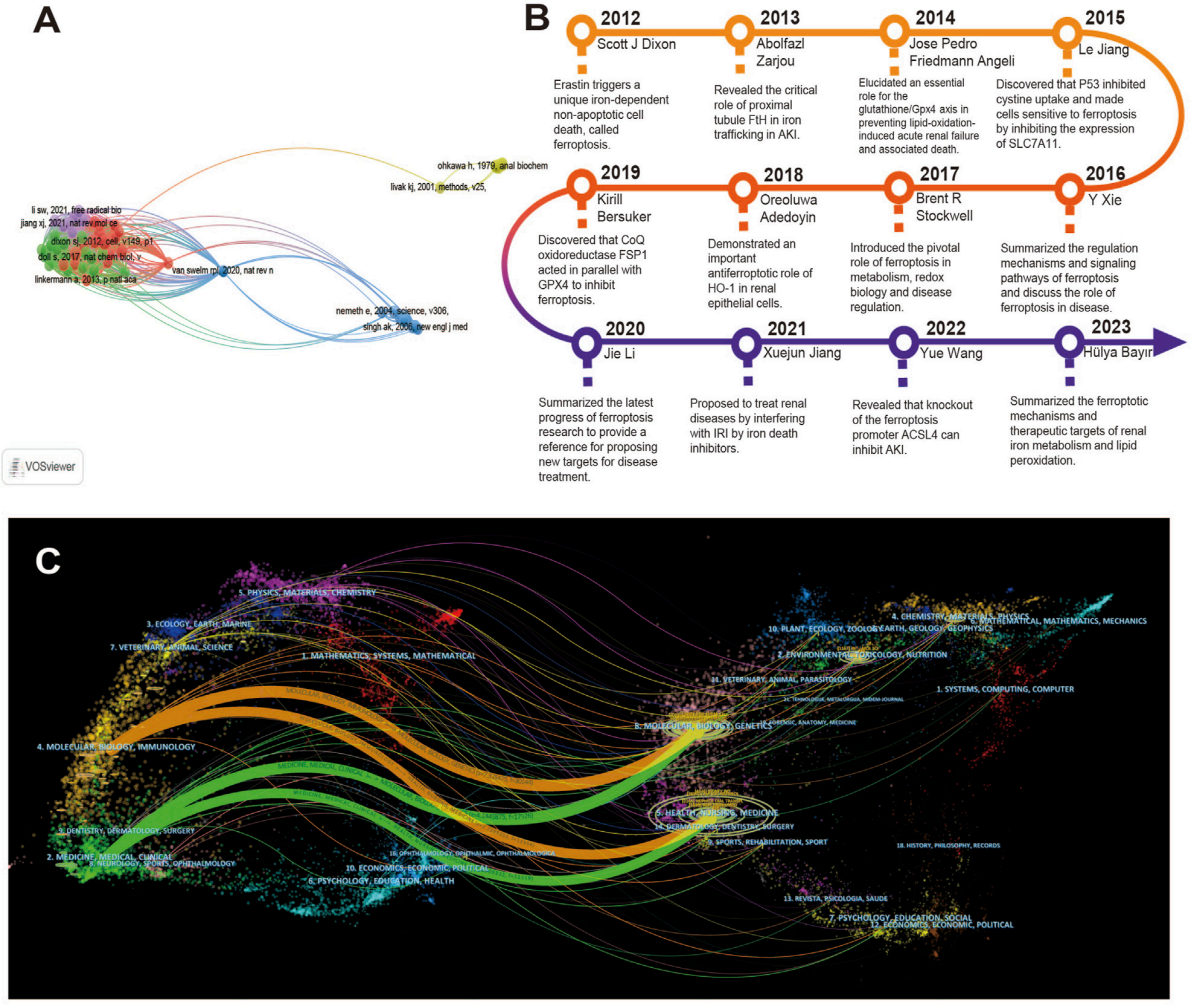
Co-cited document analysis. **(A)** Co-cited document map. Each node represents an article, and the node size is positively correlated with the number of citations. **(B)** Chronological timeline of key articles in ferroptosis in kidney disease research. **(C)** Dual map of publications (2012–2024). The citing journal distribution on the left represents relative domain applications, and the cited journals distribution on the right refers to research basis, with the curve representing citation line in the graph. The label represents the topic covered by the journal, and the color path represents the citation relationship.

The analysis of high-cited articles shows key information about specific research areas, and the number of citations reflects the importance of the article. Figure 6A shows the co-citation of articles, detailed data are shown in Table 1. The most cited article is *Ferroptosis: An Iron-Dependent Form of Non-Apoptotic Cell Death*, published by Scott J. Dixon, Kathryn M. Lemberg et al. on *Cell* in 2012. Followed by *Inactivation of the ferroptosis regulator Gpx4 triggers acute renal failure in mice*, cited 219 times, published in *Nature Cell Biology* in 2014. This article provides direct genetic evidence that the knockout of Gpx4 leads to cell death in a pathology-related form of ferroptosis, elucidates the important role of the GSH/Gpx4 axis in preventing lipid oxidation-induced acute renal failure and related death, and found an effective spiroquinoxaline derivative (Liproxstatin-1), which can inhibit ferroptosis in cells and ischemia/reperfusion-induced liver injury (Angeli et al., 2014).

**TABLE 1:** The top 15 co-cited references related to ferroptosis in kidney disease field.

NO.	Count	Author	Year	DOI	Ref.
1	153	Angeli JPF	2014	10.1038/ncb3064	Angeli et al. (2014)
2	140	Yang WS	2014	10.1016/j.cell.2013.12.010	Yang et al. (2014)
3	135	Stockwell BR	2017	10.1016/j.cell.2017.09.021	Stockwell et al. (2017)
4	125	Linkermann A	2014	10.1073/pnas.1415518111	Linkermann et al. (2014)
5	100	Martin-Sanchez D	2017	10.1681/ASN.2015121376	Martin-Sanchez et al. (2017)
6	93	Xie Y	2016	10.1038/cdd.2015.158	Xie et al. (2016)
7	90	Doll S	2017	10.1038/nchembio.2239	Doll et al. (2017)
8	81	Kagan VE	2017	10.1038/nchembio.2238	Kagan et al. (2017)
9	80	Li J	2020	10.1038/s41419-020-2298-2	Li et al. (2020a)
10	80	Yang WS	2016	10.1016/j.tcb.2015.10.014	Yang and Stockwell (2016)
11	70	Dixon SJ	2012	10.1016/j.cell.2012.03.042	Dixon et al. (2012)
12	63	Doll S	2019	10.1038/s41586-019-1707-0	Doll et al. (2019)
13	63	Bersuker K	2019	10.1038/s41586-019-1705-2	Bersuker et al. (2019)
14	61	Hou W	2016	10.1080/15548627.2016.1187366	Hou et al. (2016)
15	61	Jiang L	2015	10.1038/nature14344	Jiang et al. (2015)

In the process of sorting out the data, the papers with the highest number of co-citations each year were selected to focus on research. We summarize the main significance of these articles and construct a timeline in chronological order as shown in Figure 6B, which helps to understand the research progress of the role of ferroptosis in kidney disease (Dixon et al., 2012; Zarjou et al., 2013; Jiang et al., 2015; Jiang et al., 2021; Xie et al., 2016; Stockwell et al., 2017; Adedoyin et al., 2018; Bersuker et al., 2019; Li J. et al., 2020; Wang Y. et al., 2022; Bayır et al., 2023). The dual map of publications is shown in Figure 6C. The citing journal distribution on the left represents relative domain applications, and the cited journals distribution on the right refers to research basis, with the curve representing citation line in the graph. Through this citation relationship, the changes of hot topics can be analyzed (Chen and Leydesdorff, 2014). The existing maps show two main reference paths. The orange citation path indicates that journals published in the Molecular/Biology/Immunology field were frequently cited for research published in the Molecular/Biology/Genetic, Health/Nursing/Medicine, Dermatology/Dentistry/Surgery journals. The Green Citation Pathway shows that journals published in the fields of Medicine/Medical/Clinical, Neurology/Sports/Ophthalmology were frequently cited in research published in the journals of Molecular/Biology/Genetic, Health/Nursing/Medicine, Dermatology/Dentistry/Surgery.

### 3.6 Keywords analysis

**FIGURE 7:**
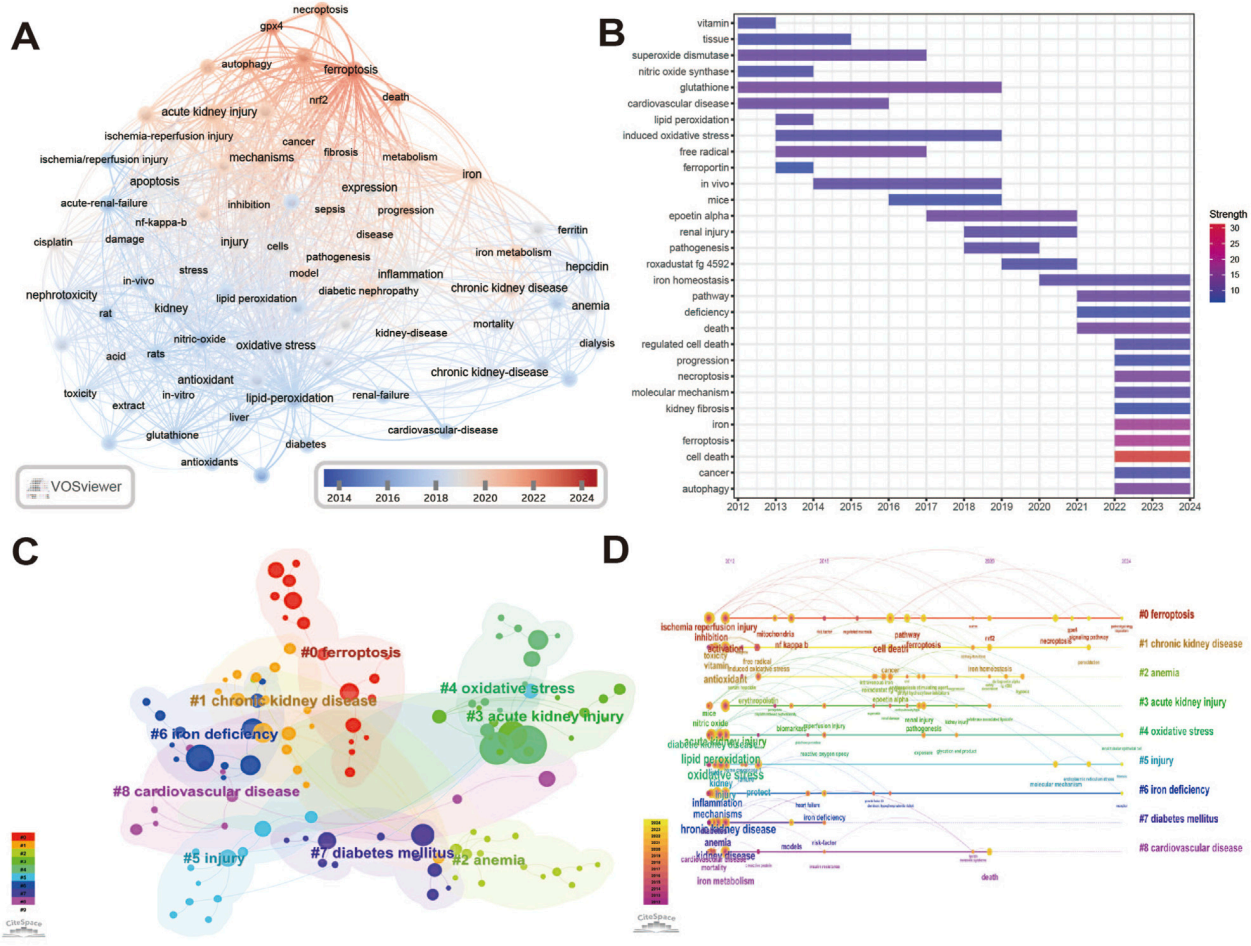
Hotspots and Frontiers of keywords. **(A)** Keyword co-occurrence graph. Each node represents a keyword, and the size of the node is proportional to the number of co-occurrences. The two co-cited keywords are connected by a curve, and the color is related to the time when the keyword appears. **(B)** Top 30 keywords with the strongest citation burst. Color segment represents the start year, end year and burst duration of the first occurrence of keywords, and color is positively correlated with the intensity of burst. **(C)** Cluster analysis of keywords. Each cluster consists of several closely related keywords, and the size of the cluster tag (#) sequence number is displayed as # 0, # 1, # 2, *etc.* **(D)** Keywords timeline diagram.

In order to further understand the research hotspots and the changes of hotspots, the analysis of keywords was carried out. The keywords in the literature are concise expressions of research topics. Visual co-occurrence analysis is helpful to detect research topics, analyze research hotspots, and monitor the rules and trends of research frontier changes in a knowledge field. High-frequency keywords often reflect research hotspots in this field. It can be seen from the keyword co-occurrence analysis (Figure 7A; Table 2) that early studies mainly focused on terms such as “glutathione”, “nitric oxide”, “vitamin”, and “lipid peroxidation”, and then transferred to “biomarker”, “inflammation”, “ckd”, and “anemia”. In contrast, contemporary research has shifted its focus to “autophagy” and “necroptosis”, marking the emergence of new research hotspots and thematic directions in this field. Figure 7B shows the outbreak of keywords. Keyword burst refers to the sudden increase in the citation intensity of a word, which is considered as an indicator map of research frontiers or emerging topics in a specific field over time (Yang et al., 2022). Glutathione, cardiovascular disease, superoxide dismutase, vitamin, nitric oxide synthase is an early subject of interest, and glutathione (2012–2019) gains the most enduring interest in the early years based on the start time and length of appearance. Autophagy, molecular mechanism, regulated cell death, cancer, progression, kidney fibrosis began to erupt in 2022 and continue to this day, indicating that these contents are the main research contents in the past 3 years. The keyword clustering graph generated based on the co-occurrence network is shown in Figure 7C, and the nine clusters formed represent the most prominent topics in this field so far (Wang Y. et al., 2024). Further analysis of clusters (Figure 7D; Table 3) shows that chronic kidney disease, oxidative stress, injury, iron deficiency have been hot research topics.

**TABLE 2:** The top 20 keywords in terms of frequency for ferroptosis in kidney disease field.

NO.	Keywords	Occurrences	NO.	Keywords	Occurrences
1	oxidative stress	915	11	kidney	229
2	lipid peroxidation	644	12	expression	212
3	aki	478	13	hemodialysis	212
4	ckd	445	14	dkd	206
5	ferroptosis	444	15	iron	205
6	inflammation	371	16	kidney disease	193
7	antioxidant	326	17	hepcidin	190
8	mechanisms	240	18	cell death	184
9	anemia	238	19	injury	163
10	apoptosis	236	20	nephrotoxicity	163

**TABLE 3:** Keywords clusters tag information.

Id	Size	Silhouette	Year	Top terms (LLR)
0	17	0.961	2017	Ferroptosis (52.5, 1.0E-4); cell death (41.49, 1.0E-4); necroptosis (31.82, 1.0E-4); ischemia-reperfusion injury (31.82, 1.0E-4); anemia (27.02, 1.0E-4)
1	16	0.98	2014	Chronic kidney disease (39.17, 1.0E-4); nephrotoxicity (32.48, 1.0E-4); ferroptosis (21.8, 1.0E-4); oxidative stress (20, 1.0E-4); potassium bromate (15.01, 0.001)
2	15	0.986	2018	Anemia (45.46, 1.0E-4); hypoxia-inducible factor (35.64, 1.0E-4); meta-analysis (27.94, 1.0E-4); roxadustat (27.94, 1.0E-4); daprodustat (24.91, 1.0E-4)
3	14	0.988	2015	Acute kidney injury (75.32, 1.0E-4); renal toxicity (33.11, 1.0E-4); reperfusion injury (33.11, 1.0E-4); nitric oxide (30.25, 1.0E-4); sepsis (26.32, 1.0E-4)
4	14	1	2014	Axidative stress (99.08, 1.0E-4); diabetic nephropathy (93.8, 1.0E-4); lipid peroxidation (57.8, 1.0E-4); hepcidin (34.9, 1.0E-4); anemia (28.83, 1.0E-4)
5	12	0.893	2015	Injury (30.92, 1.0E-4); protect (20.13, 1.0E-4); chronic kidney disease (15.49, 1.0E-4); iron metabolism (11.97, 0.001); nitric oxide synthase (11.64, 0.001)
6	12	0.959	2014	Chronic kidney disease (120.41, 1.0E-4); iron deficiency (53.74, 1.0E-4); hepcidin (52.75, 1.0E-4); anemia (49.72, 1.0E-4); erythropoietin (22.79, 1.0E-4)
7	11	0.943	2012	Diabetes mellitus (40.07, 1.0E-4); kidney disease (17.51, 1.0E-4); acute kidney injury (16.4, 1.0E-4); iron deficiency anemia (15.33, 1.0E-4); hepcidin (12.09, 0.001)
8	11	0.979	2014	Cardiovascular disease (23.51, 1.0E-4); iron metabolism (21.77, 1.0E-4); metabolic syndrome (20.7, 1.0E-4); nephrotoxicity (16.36, 1.0E-4); stage renal disease (14.8, 0.001)

## 4 Discussion

### 4.1 Summary of findings

In the past 12 years, academic interest and research on ferroptosis in kidney disease have increased significantly, making the number of related studies increasing every year. Before 2018, the number of published papers was relatively stable and limited. From 2018 to 2021, the number of papers has increased significantly, indicating that the scope and depth of research are expanding. After 2021, it has shown a rapid growth trend, with an average annual growth of about 73 articles. This trend highlights the research potential of ferroptosis in kidney disease and attracts more scholars ' attention in the future.

The United States and China are in a leading position in the field of ferroptosis research in kidney diseases. China has the largest number of publications, and artisans have the highest number of citations, far ahead of other countries/regions. However, the cooperation between different countries is still insufficient, which is characterized by small distribution and low frequency of cooperation. It is necessary to call on more countries and corresponding institutions to break the current situation and actively form strong cooperation to further promote the in-depth development of this field. The most productive author is Andreas Linkermann who published 23 articles from 2012 to 2024 from Dresden University of Technology. Andreas Linkermann is an authoritative scholar in the fields of cell biology, biochemistry and molecular biology, urology and nephrology. *Ferroptosis: An Iron-Dependent Form of Non-Apoptotic Cell Death* is the most cited article in the analysis of ferroptosis in kidney disease. This study found and gave the definition and characteristics of ferroptosis, indicating that ferroptosis is triggered by the oncogenic RAS selective lethal small molecule erastin. Moreover, small molecule siderostatin-1 was identified as an effective inhibitor of ferroptosis in cancer cells and glutamate-induced cell death in organotypic rat brain sections (Dixon et al., 2012). The change of the highest cited literature each year reflects the gradual deepening process of ferroptosis research. With the discovery of more and more molecules and pathways, the number of action sites that can be selected as targets has also increased, providing a richer therapeutic strategy.

In the top 20 cited publications, 13 of them were experimental articles and seven were reviews, indicating that the role of ferroptosis in kidney diseases has a lot of preliminary basis. Jose Pedro et al. found that inactivation of the ferroptosis regulator Gpx4 triggers acute renal failure in mice. Ferroptosis is triggered by extra-mitochondrial lipid peroxidation and is characterized by the release of oxidized lipid mediators. Inducible knockout of Gpx4 leads to increased lipid peroxidation (Angeli et al., 2014). Wan Seok Yang We et al. made a new identified here a ligand-binding site on GPX4 and determined the specific lipids oxidized during ferroptosis. Two key drivers of lipid peroxidation during ferroptosis, which were lipoxygenases and phosphorylase kinase G2 were identified (Yang et al., 2016). In GPX4 deficiency in mouse embryonic fibroblasts (Pfa1) cells, lipid hydroperoxides accumulate in ferroptotic endoplasmic reticulum and further discovery whereby lipoxygenases (LOX) generate doubly- and triply-oxygenated (15-hydroperoxy)-di-acylated phosphatidylethanolamines (PE) species which act as death signals. PE as only one class of phospholipids that undergos oxidation in the ER-associated compartments with the specificity towards two fatty acyls–arachidonoyl (AA) and adrenoyl (AdA) (Kagan et al., 2017). Sebastian Doll et al. used an expression cloning approach to identify genes in human cancer cells that are able to complement the loss of GPX4. They found that ferroptosis suppressor protein 1 (FSP1) which was initially described as a pro-apoptotic gene, confers protection against ferroptosis elicited by GPX4 deletion and further demonstrate that ferroptosis suppression by FSP1 is mediated via ubiquinone (CoQ10): its reduced form ubiquinol traps lipid peroxyl radicals that mediate lipid peroxidation, while FSP1 catalyses its regeneration by using NAD(P)H. In conclusion, FSP1/CoQ10/NAD(P)H exists as a stand-alone parallel system, which co-operates with GPX4 and glutathione (GSH) to suppress phospholipid peroxidation (pLPO) and ferroptosis (Doll et al., 2019).

The analysis of keywords reflects the focus of ferroptosis in kidney diseases. “Oxidative stress” (915) and “lipid peroxidation” (644) are the most important characteristics of ferroptosis, which are the main objects of early research. Subsequently, “biomarkers” (74), “inflammation” (371) and “ckd” (445) were the focus of attention, and the exploration of the mechanism of ferroptosis in the process of kidney disease was gradually enriched. Subsequent studies will focus on “gpx4” (88), “autophagy” (71), “necroptosis” (65) and “ferroptosis” (444), hoping to develop new targets in these pathways that have been explored and broaden the perspective of the treatment of kidney diseases. The results of keyword bursts showed that glutathione (2012–2019), superoxide dismutase (2012–2017), ferroportin (2013–2014), roxadustat (2019–2021), iron (2022–2024) are important biomarkers that have gradually emerged since 2012.

In conclusion, ferroptosis is an emerging field of research, the mechanism of which is not yet fully understood, but has shown potential in the treatment of a variety of diseases. In the future, with further in-depth research into the mechanism of iron death, more effective treatment options may be developed.

### 4.2 Novel biomarkers

Due to the lack of specific markers, ferroptosis has been difficult to identify under physiological conditions (Dixon et al., 2012). In the kidney, the reabsorption of iron ions occurs in the renal tubules after glomerular filtration, and the renal tubular epithelial cells are extremely active sites for iron ions and ROS, which makes the kidney extremely sensitive to ferroptosis (Kim et al., 2021). More and more studies have found that ferroptosis is a key driver of various kidney diseases, such as acute kidney injury (AKI), diabetic kidney disease, and ischemia/reperfusion injury (Feng et al., 2023; Wang et al., 2023). In the past 10 years, the research mainly focused on the mechanism of ferroptosis regulation pathway. However, the most important thing is to translate the mechanism of ferroptosis researched into clinical practice. Therefore, Kamyar et al. proposed that the identification of biomarkers for ferroptosis may help to make full use of ferroptosis research in the past decade and transform ferroptosis biology into new diagnostic or therapeutic methods (Hadian and Stockwell, 2021).

With the advancement of sequencing technology and bioinformatics, machine learning models have been widely used in the screening of disease diagnosis marker genes and have shown good results. In a study based on bioinformatics to explore new ferroptosis gene biomarkers in diabetic nephropathy (DN), it was found that the six ferroptosis-related biomarkers (ALOX5, CCL5, KRT19, LCN2, LTF, and RRM2) identified were closely related to the immune status of DN and may play an important role in the pathogenesis of DN (Huang and Yuan, 2024). Arachidonate 5-lipoxygenase (ALOX5), as a key enzyme mediating lipid peroxidation catalyzes the peroxidation of polyunsaturated fatty acids. Inhibition of ALOX5 nuclear translocation and its phosphorylated expression can effectively reduce the level of lipid peroxidation, thereby preventing the occurrence of ferroptosis (Li et al., 2023). Lipocalin-2 (LCN2), also known as neutrophil gelatinase-associated lipocalin (NGAL), is a small circulating protein that is highly regulated in a variety of pathological conditions, making it a useful biomarker for various disease states (Buonafine et al., 2018). LCN2 is an effective iron chelating agent, but homodimer variants that are significantly upregulated in certain pathological conditions cannot chelate iron, which may promote the downstream toxic effects of iron accumulation (Golonka et al., 2019). Studies have shown that LCN2 is positively correlated with albumin excretion rate and hemoglobin levels (Zachwieja et al., 2010). SY Kim et al. found that the level of NGAL in patients with severe albuminuria was significantly increased, which was moderately correlated with the degree of albuminuria. The level of NGAL can independently reflect the degree of renal tubular injury in DN patients (Kim et al., 2018). NGAL is expected to be a potential biomarker for DN. In recent years, with the increase of research, more biomarkers have appeared. In 2022, Jiang Y et al. found a tumor-suppressive circular RNA circLRFN5 and identified its role in ferroptosis, making it a promising target for ferroptosis-dependent therapy (Song et al., 2020). Su J et al. found that single-stranded DNA binding protein 1 (SSBP1) was significantly upregulated when ferroptosis occurred to protect cells and could be used as a therapeutic target and biomarker for ferroptosis (Doll et al., 2019).

Recently, it is still a challenging task to find specific biomarkers of ferroptosis. A new study have shown that hyperoxidized peroxiredoxin (PRDX) 3 is identified as the first specific marker of ferroptosis (Cui et al., 2023). PRDX3 is a mitochondrial-specific peroxidase and a member of the antioxidant enzyme family PRDXs (Lee, 2020). PRDX3 can be overoxidized due to exposure to mitochondrial lipid peroxides. However, PRDX3 is only overoxidized during ferroptosis and not in other cell death pathways. Subsequently, overoxidized PRDX3 translocated from mitochondria to plasma membrane, and the presence of hyperoxidized PRDX3 on the plasma membrane inhibited the uptake of cystine, thereby promoting ferroptosis (Cui et al., 2023). In addition, some scholars have found that high glucose stimulation increases the acetylation of PRDX3, which in turn promotes the excessive oxidation of PRDX3 and leads to pancreatic β-cell dysfunction (Chen et al., 2008; Lee, 2020). Jun Jian et al. found that the expression of PRDX3 in fibrotic kidneys was significantly lower than that in adjacent tissues in human kidney specimens. Subsequently, overexpression of PRDX3 *in vivo* and *in vitro* models revealed that Unilateral Ureteral Obstruction (UUO) and transforming growth factor (TGF)-β1 induced ROS production, fibrosis-related factors and inflammatory cytokines were significantly reduced, and PRDX3 knockdown mice were more prone to UUO-induced renal fibrosis (Jian et al., 2024). The results of this study provide a long-awaited ferroptosis marker for the field of ferroptosis, enabling more accurate detection of this unique form of cell death.

### 4.3 Mechanisms

The research on the mechanism of ferroptosis has mainly shifted from iron metabolism disorders, lipid peroxide accumulation to the crosstalk of disease-related signaling pathways such as autophagy, necroptosis with ferroptosis. This coincides with our results. Recent studies have confirmed the important role of ferroptosis in the pathophysiology of various kidney diseases and become a new research focus in the field of renal fibrosis (Long et al., 2024).

Intracellular iron overload and lipid ROS species accumulation are the key links in ferroptosis. Any factor affecting the balance of iron metabolism and redox system may induce ferroptosis (Li J. et al., 2020). The Xc-GSH-GPX4 axis is an important antioxidant defense system in cells. Gpx4 acts as an inhibitory protein in lipid peroxidation, which reduces lipid hydroperoxides to lipid alcohols to prevent ROS accumulation. The activity of Gpx4 is regulated by GSH, and the level of intracellular GSH is mainly regulated by system Xc^−^, which mediates the uptake of cystine and then converts it into cysteine that synthesizes GSH (Kim et al., 2021). Studies have shown that the expression of Gpx4 in renal biopsy samples of DKD patients was significantly reduced (Kim et al., 2021). Renal tubular injury is a key factor in the development of DKD. High glucose levels can trigger iron overload, decreased antioxidant capacity, excessive ROS production and lipid peroxidation in renal tubular cells (Wang W.-J. et al., 2021). Recently, Wang et al.found that there is a close relationship between Gpx4 and DKD, including urinary protein, Scr, eGFR and the percentage of glomerulosclerosis in renal specimens, suggesting that Gpx4 level may be an independent predictor of renal outcome in tubulointerstitium (Wang Y.-H. et al., 2022). Another study showed that in the db/db mouse model, the expression level of Acyl-CoA synthetase long-chain family (ACSL4) increased and the expression level of Gpx4 decreased, while the lipid peroxidation products and iron content of DN mice increased (Wang et al., 2020). The above evidence suggests that the Xc-GSH-GPX4 axis, especially Gpx4, may be a key link in the progression of ferroptosis in DN.

In recent years, the crosstalk between ferroptosis and other cellular processes in kidney disease has been paid more and more attention. Studies have shown that autophagy is involved in the regulation of ferroptosis through the degradation of proteins and organelles, thereby controlling iron accumulation and lipid peroxidation (Hou et al., 2016). Several studies have shown that nuclear receptor coactivator 4 (NCOA4)-ferritin autophagy has a significant effect on ferroptosis. The depletion of NCOA4 reduced the accumulation of intracellular free iron and lipid peroxides, resulting in reduced ferroptosis (Mancias et al., 2014). Fei Deng et al. reported that in patients with acute tubular necrosis, significant changes in ferritin phagocytosis biomarker NCOA4 were observed. Overexpression of inositol oxidase (MIOX) can promote NCOA4-mediated ferritin autophagy and cisplatin-induced ferroptosis in AKI cells (Liu et al., 2018). A large number of studies have shown that necroptosis is associated with AKI and acute tubular necrosis (Ratliff et al., 2016). Wulf Tonnus et al. found that Gpx4 or ferroptosis suppressor protein 1 (FSP1) dysfunction is a unique form of renal tubular necrosis. Subsequently, this team specifically designed and developed a single small molecule necrostatin-1f (Nec-1f) that can simultaneously inhibit necroptosis and ferroptosis. The results showed that Nec-1f simultaneously inhibited the expression of necroptosis protein receptor interacting protein kinase (RIPK) one and ferroptosis, and its possible mechanism may be related to the dual role of the RIPK family (Tonnus et al., 2021). Studies have shown that RIPK3 not only phosphorylates mixed lineage kinase domain-like protein (MLKL) to induce necroptosis, but also phosphorylates FSP1 to inhibit its enzyme activity, thereby promoting ferroptosis (Lai et al., 2024). In addition, when multiple cell death programs are simultaneously activated, the inhibition of a single molecular pathway is an ineffective method of preventing cell death, rather, it may alter the manner in which cell death occurs. For example, MLKL deficiency in cultured fibroblasts prevents necroptosis, but it is beneficial to cystine deprivation-induced ferroptosis. The lack of ferroptosis protein ACSL4 prevents ferroptosis, but it is beneficial to the occurrence of necroptosis induced by tumor necrosis factor and pan-caspase inhibition (Müller et al., 2017). The potential for multiple modes of cell death to act synergistically with ferroptosis to mediate disease progression is a possibility that warrants further investigation.

### 4.4 Therapeutic strategies

As the role of ferroptosis in the occurrence and development of various kidney diseases has been gradually recognized, it has become a research hotspot to alleviate the progression of diseases by inhibiting or activating ferroptosis. A variety of natural and synthetic drugs related to ferroptosis have been found, including inducers and inhibitors. Renal tubular iron deposition is a common feature of CKD patients. In animal models, iron chelators deferoxamine, desferoxand and cicloprox can prevent cisplatin-induced AKI or 5/6 nephrectomy-induced CKD (Ikeda et al., 2021; Wang J. et al., 2022). Studies have shown that the ferroptosis inhibitor Liproxstatin-1 (Lip-1) reduces UUO-induced renal fibrosis by inhibiting ferroptosis-mediated tumor endothelial cells (TECs) death. The experimental results confirmed that it can reduce the expression of pro-fibrotic factors in the UUO model and ferroptosis in HK2 cells, and improve renal histopathological abnormalities in mice (Zhang et al., 2021). Friedmann et al. found that Lip-1 prevented both RSL3-induced primary human proximal TEC death and Gpx4 deficiency-induced acute renal failure (Angeli et al., 2014). Studies have found that ferroptosis inducers reduce Gpx4 activity and enhance intracellular lipid peroxidation, further exacerbating the progression of renal injury and fibrosis. The use of ferroptosis inhibitors tocilizumab, formononetin and Tectorigenin can prevent the development of renal fibrosis by inhibiting ferroptosis-related lipid peroxidation and GSH consumption (Doll et al., 2017). A recent study by Li et al. also reported that roxadustat (FG-4592) pretreatment inhibited ferroptosis in renal tubular epithelial cells through the Akt/GSK3β/Nrf2 signaling pathway in folic acid-induced AKI, thereby improving renal injury and renal fibrosis (Li X. et al., 2020). N-acetylcysteine (NAC) has been developed as a cysteine donor to supplement GSH and is considered to be an antioxidant. A study found that low concentrations of NAC can reduce Erastin-induced proximal tubular ferroptosis (Ratliff et al., 2016). In 2022, a study found that NAC combined with insulin administration upregulated the level of SLC7A11 mRNA in the kidney and increased the intracellular GSH concentration by nearly 4 times, thereby reducing renal fibrosis and improving renal function (Li et al., 2022). Clinical drugs thiazolidinediones play a renal protective role by reducing lipid peroxidation, redox stress and inflammatory response. Rosiglitazone can reduce the level of arachidonic acid, similar to the level of ACSL4 knockout cells, and also improve the survival rate of Gpx4 knockout mice to avoid renal failure death (Doll et al., 2017). Treatment with dapagliflozin significantly improved the ferroptosis environment in DKD by attenuating excessive activation of the HIF-1α/HO1 axis *in vivo* and *in vitro* (Wang Y.-H. et al., 2024).

Current research is continuing to explore the specific regulatory mechanisms of ferroptosis and how to intervene in the iron death process by regulating iron metabolism and antioxidant systems. For example, regulating the expression of relevant genes through gene editing technology, or developing drugs that can inhibit iron death, in the hope of providing new treatment strategies for related diseases. In our results, two of the top 20 citations related to the treatment of ferroptosis. Rachid Skouta et al. found that ferrostatins inhibit oxidative lipid damage and cell death in diverse disease models. The mechanism by which it exerts therapeutic effects involves the depletion of specific membrane lipids, most likely due to oxidative destruction (Skouta et al., 2014). Chinese scholar found that quercetin (QCT) significantly inhibited both Era and RSL3 induced-ferroptosis, increased the cell viability and decreased cellular lipid ROS. Meanwhile, QCT also inhibited the chemotaxis of macrophages and inflammatory response induced by ferroptosis in AKI. As expected, QCT also showed as a therapeutic drug candidate for both I/R-AKI and FA-AKI, protecting from functional acute renal failure and structural organ damage (Wang Y. et al., 2021). Interestingly, QCT is also a main component of “Huangkui capsule”, which was proven to have wide renal protective effect and is widely used for DN and other kidney diseases in China (Cai et al., 2017; Yang et al., 2019). The inhibitory effect of QCT on ferroptosis of kidney tubular epithelial cells shown here might provide the possible mechanism for “Huangkui capsule”. Platycodin D inhibits ferroptosis in high glucose-induced cells by regulating GPX4 expression (Huang et al., 2022, p. 4). Glabridin treatment can antagonize oxidative stress and ferroptosis in DN by down-regulating the expression of SLC7A11 and SLC3A2 *in vivo* and *in vitro* (Tan et al., 2022). In summary, these findings indicate that ferroptosis is a key factor in the progression of kidney disease, and targeting ferroptosis may be a promising strategy for the treatment of renal fibrosis.

## 5 Conclusion

Our study provide comprehensive information on the publication performance of ferroptosis in kidney disease through the bibliometric analysis. An increasing number of studies have demonstrated the potential of ferroptosis as a therapeutic strategy for kidney disease treatment, particularly through the accumulation of lipid peroxidation. On this hot topic, more and more scholars, institutions and countries have poured in and published a large number of high-quality works. The collaborative efforts of institutions and researchers across countries have played a vital role in advancing research within this domain. It is imperative that institutions and researchers from diverse nations collaborate to identify relevant areas of focus and enhance our comprehension of this evolving and innovative subject matter. This evolving landscape highlights ferroptosis is a critical area of study, indicating that it will remain to be a central focus for future research. With the assistances of the continued interdisciplinary collaboration and research, researchers will unravel the complexities of ferroptosis and its impact on disease mechanisms and treatments in the future.
